# Immuno-digital invasive cleavage assay for analyzing Alzheimer’s amyloid ß-bound extracellular vesicles

**DOI:** 10.1186/s13195-022-01073-w

**Published:** 2022-10-03

**Authors:** Kohei Yuyama, Hui Sun, Yasuyuki Igarashi, Kenji Monde, Takumi Hirase, Masato Nakayama, Yoichi Makino

**Affiliations:** 1 Faculty of Advanced Life Science, Hokkaido University, Kita-21, Nishi-11, Kita-ku, Sapporo, 001-0021 Japan; 2 Technical Research Institute, TOPPAN INC., 4-2-3 Takanodaiminami, Sugito-machi, Saitama 345-8508 Japan

**Keywords:** Digital ICA, Extracellular vesicles, Amyloid-ß protein, Ganglioside GM1, Biomarker, Alzheimer’s disease

## Abstract

**Background:**

The protracted preclinical stage of Alzheimer’s disease (AD) provides the opportunity for early intervention to prevent the disease; however, the lack of minimally invasive and easily detectable biomarkers and their measurement technologies remain unresolved. Extracellular vesicles (EVs) are nanosized membrane vesicles released from a variety of cells and play important roles in cell–cell communication. Neuron-derived and ganglioside-enriched EVs capture amyloid-ß protein, a major AD agent, and transport it into glial cells for degradation; this suggests that EVs influence Aß accumulation in the brain. EV heterogeneity, however, requires the use of a highly sensitive technique for measuring specific EVs in biofluid. In this study, immuno-digital invasive cleavage assay (idICA) was developed for quantitating target-intact EVs.

**Methods:**

EVs were captured onto ganglioside GM1-specific cholera toxin B subunit (CTB)-conjugated magnetic beads and detected with a DNA oligonucleotide-labeled Aß antibody. Fluorescence signals for individual EVs were then counted using an invasive cleavage assay (ICA). This idICA examines the Aß-bound and GM1-containing EVs isolated from the culture supernatant of human APP-overexpressing N2a (APP-N2a) cells and APP transgenic mice sera.

**Results:**

The idICA quantitatively detected Aß-bound and GM1-containing EVs isolated from culture supernatants of APP-N2a cells and sera of AD model mice. The idICA levels of Aß-associated EVs in blood gradually increased from 3- to 12-month-old mice, corresponding to the progression of Aß accumulations in the brain of AD model mice.

**Conclusions:**

The present findings suggest that peripheral EVs harboring Aß and GM1 reflect Aß burden in mice. The idICA is a valuable tool for easy quantitative detection of EVs as an accessible biomarker for preclinical AD diagnosis.

**Supplementary Information:**

The online version contains supplementary material available at 10.1186/s13195-022-01073-w.

## Background

Amyloid β-protein (Aβ) accumulation and deposition in the brain are early and consistent pathological hallmarks of Alzheimer’s disease (AD). The glycosphingolipid ganglioside GM1 is linked to Aß-associated pathology in AD [1, 2]. GM1 is found in the outer layer of cell membranes, with their glycans exposed to the external milieu. GM1 moves laterally across the membranes and forms high-density clusters, which monomeric Aß recognizes and then binds to [3]. GM1 also promotes the assembly of Aß into amyloid fibrils [4]. Aß–GM1 complexes are found in the brain tissues and interstitial fluid of aged monkeys and patients with AD [5, 6]. Glycosphingolipids, including GM1, are also localized in extracellular vesicle (EV) membranes [7]. EVs are heterogeneous populations of membrane vesicles released from various cells [8]. Our earlier findings using quantitative glycomics show that GM1 is more abundant in EVs than in their parental cells and that concomitantly Aß binds to EVs through a part of the GM1 domains [9–11]. Aß-bound and GM1-containing EVs released from neurons are internalized and degraded by microglia, suggesting that the neuronal EVs may contribute to Aß clearance. In the presence of an excess of EVs or the absence of glial phagocytic activity for these membrane vesicles, EVs may, therefore, promote Aß accumulation and amyloid plaque formation [7, 12].

Liquid biopsy, which is based on the analysis of circulating biological molecules such as nucleic acid, proteins, and metabolites, is a powerful tool for determining the pathological stages of several diseases such as cancer and dementia [13, 14]. This technique is used for the molecular profiling and diagnostic imaging of biopsy tissue for evaluating pathogenesis and disease progression [15]. EVs are target candidates for liquid biopsy; they possess the characteristic profiles of cargo proteins, mRNA, miRNA, and surface lipids that vary depending on parental cell types and conditions. Moreover, EVs are stably circulated in almost all bodily fluids, including blood, cerebral spinal fluid, and urine. EVs, therefore, are potential non-invasive biomarkers for the pathogenesis and prognosis of a wide range of diseases, including cancers, heart disease, obesity, and dementia [16–18]. Aß is found in CSF- and serum-derived EVs in amyloid precursor protein transgenic (APP Tg) mice [9–11].

Recent technological advances facilitated by microfluidics have enabled us to make significant progress in our capacity to analyze liquid biopsy molecules [19]. In particular, one of the most extensively used applications is a droplet-based digital bioassay system that uses microcompartalization to detect single molecules. Immuno-digital invasive cleavage assay (idICA) allows femtogram-level measurements of target proteins [13]. In the present study, we developed a novel idICA system for the quantitative detection of EVs harboring GM1 and Aß in the culture supernatant of APP-N2a cells and sera from AD model mice (APP Tg mice). We, furthermore, evaluated the potential of Aß-bound and GM1-containing EVs in serum-derived EVs in APP Tg mice at different ages.

## Methods

### Isolation of EVs from culture supernatants

Murine neuroblastoma Neuro2a (N2a) cells, human APP751-stable transfected N2a cells, and HEK293 cells were maintained in Dulbecco’s modified Eagle’s medium (Invitrogen, Carlsbad, CA) supplemented with 10% fetal bovine serum. One day before EV isolation, the cells were transferred to a serum-free medium. EVs were collected from cell supernatants following differential ultracentrifugation. The supernatants were sequentially centrifuged at 2000 × *g* for 10 min, 10,000 × *g* for 30 min to remove cells and debris, and then 100,000 × *g* for 1 h to pellet the EVs. EV protein content was measured with the BCA assay (Nacalai Tesque, Kyoto, Japan). Sizes and densities of the EVs suspended in PBS were analyzed with a qNano system (Izon Science, Cambridge, MA) using NP200 nanopores and qNano Izon analysis software. CPC100 was used as the calibration sample in this study. Microphotographs of the EVs were obtained with an HD-2000 scanning transmission electron microscope (Hitachi, Tokyo, Japan).

### Mouse serum collection

Heterozygotic transgenic mice expressing human APP harboring the Swedish and Indiana (KM670/671NL, V717F) mutations (APP_SweInd_, MMRRC_034836-JAX) were from Jackson Laboratory (Bar Harbor, ME) housed in a room kept at 23 ± 1°C with a 12- h light/dark cycle and allowed free access to tap water and food (AIN-93M). The animals were housed in a room (temperature, 23 ± 1 °C) with a 12 h light/dark cycle and allowed free access to tap water and food (AIN-93M). Animal protocols were approved by the Hokkaido University Animal Care Committees; all experiments followed the guidelines and regulations of this committee. Blood (0.5–1 mL) was collected from the heart of each mouse and incubated at 4 °C for 13 h. After centrifugation at 1000 × *g* for 20 min, serum supernatants were collected individually or mixed uniformly with the same volume of serum supernatants within groups. All samples were stored at − 80 °C before use.

### Isolation of EVs from mouse sera

Mouse sera were centrifuged at 2000 × *g* for 10 min and then at 10,000 × *g* at 4 °C for 30 min to remove cells and debris. EVs were then purified using the MagCapture^TM^ Exosome Isolation Kit PS (FUJIFILM Wako Pure Chemical Corporation, Osaka, Japan), according to the manufacturer’s instructions. Purified EVs were centrifuged at 100,000 × *g* at 4 °C for 1 h; pelleted EVs were then resuspended in PBS. EV particle size and number were analyzed using the qNano system (Izon Science).

### Device fabrication

The digital devices were fabricated by sticking a cover component to a part of the microwell. Briefly, hydrophobic reaction spaces were created using COPs to construct the microwell parts. The COP well had a diameter of 5 μm, allowing it to efficiently trap a target bead (diameter, 3 μm) into a well. On one device, there were 100 blocks, each with 10,000 wells corresponding to 10^6^ reaction fields on the device. The cover component was equipped with COP-shaped injection and efflux ports and was attached by pulse laser welding to a microwell component to form a 50-μm flow pass between them.

### Immuno-digital ICA

#### Preparation of cholera toxin B (CTB)-coated capture beads

To couple CTB (FUJIFILM Wako) to magnetic beads, 100 μg of carboxyl group-modified magnetic beads (Magnosphere MS300/Low Carboxyl, JSR, Tokyo, Japan) in 0.1 M MES (pH 6.0) were incubated with 0.5 μg CTB at room temperature for 30 min with rotation. Then, 10 μL of 10 mg/mL 1-ethyl-3-(3-(dimethylaminopropyl)carbodiimide hydrochloride (EDC) in 0.1 M MES (pH 6.0) buffer was added into the mixture and rotated at room temperature for 3 h. CTB-coupled beads were then blocked with 0.5% bovine serum albumin (BSA), washed twice with PBST (0.1% Tween20/PBS), and resuspended in 100 μL of 0.1% Tween-20/PBS (PBST) until use.

#### Preparation of a DNA oligo-labeled detection antibody

Using a protein-oligo conjugation kit (Solulink Bioscience, San Diego, CA), detection antibodies [anti-CD9 (R&D Systems, Minneapolis, MN) or anti-human Aß (BAN50, FUJIFILM Wako)] were modified at available lysine residues, to which an S-HyNic was conjugated. Simultaneously, a DNA oligonucleotide (IL28-t or IL28-c) was covalently modified with a 4-FB at the 5′ -end. The 4-FB-modified oligonucleotide and S-HyNic-conjugated antibody were mixed at a ratio of 20 oligos to 1 antibody; the N-hydroxysuccinimide (NHS) ester reaction occurred in the presence of the Turbolink^TM^ catalyst. Excess oligo was removed from the antibody-oligo conjugates with molecular weight cut-off spin columns.

#### Beads trapping and ICA

Fifty μL of 1 mg/mL Magnosphere LC-300, coated with CTB, EVs, and 5 μL of 20 ng/mL IL28t or IL28c-detection antibody conjugates in 0.5% BSA/PBST, were mixed up to a total volume of 100 μL and incubated with rotation for 1 h at room temperature to form the bead–EV–antibody complex. The complex was washed five times with PBST, resuspended in 20 μL PBS, and introduced into the digital counting device through the access port. Following the subsequent addition of 20 μL ICA reaction solution, the device was finally enclosed by the slow injection of 100 μL Fluorinert^TM^ FC-40 immiscible fluorocarbon oil. For the ICA reaction, the digital device was then incubated at 66 °C for 15 min. Bright-field and fluorescence images of the device were captured with a fluorescence microscope BZ-X710 (Keyence, Osaka, Japan); beads and fluorescence signals with beads in microwells were recognized and counted using BZ-H3A Analyzer software (Keyence). Statistical analysis and line fitting were performed by GraphPad Prism 8.4.3 (San Diego, CA).

### Western blot analysis

Western blot analysis was performed according to the standard Laemmli-SDS-PAGE methods. Protein samples were boiled for 5 min under reducing conditions. Monoclonal antibodies against CD9 (MAB5218, R&D Systems, Minneapolis, MN), Aß (BAN50, FUJIFILM, Tokyo, Japan), and ßIII tubulin (#2146, Cell Signaling, Danvers, MA) were used. Ganglioside GM1 was detected by horseradish peroxidase-conjugated CTB (Sigma-Aldrich, Burlington, MA) [9, 20]. Protein bands were visualized using a combination of an ECL Plus kit (GE Healthcare) and an LAS4000 imaging system (FUJIFILM).

### Electron microscopy

Isolated N2a cells-derived EVs and 12-month-old APP Tg mouse serum-derived EVs were resuspended in TBS and applied to a collodion-covered grid and negatively stained with 2% phosphotungstic acid (Nisshin EM, Tokyo, Japan). Microphotographs were obtained with an HD-2000 scanning transmission electron microscope (Hitachi).

### Immunohistochemistry

Brain tissues were fixed with 4% paraformaldehyde in PBS. Thirty-micrometer-thick brain sections were blocked with 5% BSA and immunostained with a monoclonal antibody against Aß (6E10), following brief treatment with formic acid. Aß signals were visualized using the ABC elite kit (Vector Laboratories). Confocal images were obtained with a Keyence fluorescence microscope BZ-X710.

### Aß ELISA

Aß_1-40_ and Aß_1-42_ levels were determined with a sandwich ELISA (FUJIFILM Wako). Mouse brains were homogenized in 4 M guanidine-HCl buffer (pH 8.0) with an ultrasonic homogenizer (TAITEC, Saitama, Japan). The homogenates were incubated at room temperature for 3 h, diluted in 0.1% BSA in PBS, and centrifuged at 16,000 × *g* for 20 min. The supernatants were collected and used for the ELISA. Mouse sera were used directly in the ELISA, and all samples used were measured in duplicate.

## Results

**Fig. 1:**
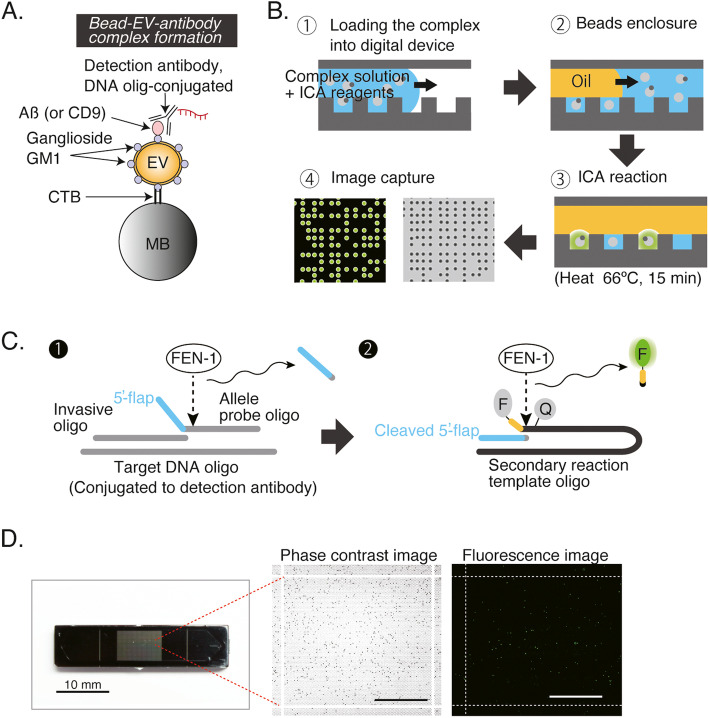
Immuno-digital ICA (idICA) for counting Aß-bound EVs. **A**, **B** Workflow of immune-digital ICA analysis of Aß-bound EVs. Ganglioside GM1-containing EVs are captured by cholera toxin B subunit (CTB)-coated magnetic beads (MB) and then reacted with the DNA oligo-conjugated detection antibody against an exosome marker protein CD9 or Aß (**A**). The resultant EV–bead–antibody complex in **A** and substrates for ICA are loaded into the digital device, enclosed into individual microwells by fluorinated oil, and analyzed by fluorescent imaging after the ICA reaction at 66 °C for 15 min (**B**). **C** Schematic illustration of ICA. (1) Invasive oligonucleotides and probe oligonucleotides hybridize to target DNA and generate 5′-flap structures in the probe oligonucleotides, which are cleaved by FEN-1. The target DNA is conjugated to detection antibodies. (2) The cleaved 5′-flaps bind to fluorescent probes and form 5′-flap structures between a quencher molecule [Q] and a fluorophore [F]. Cleavage of 5′-flaps by FEN-1 emits fluorescence signals. Unannealed 5′-flaps are shown in blue and yellow. Arrows indicate 5′-flap cleavage by FEN-1. **D** The digital device used in this study. There are 100 blocks of well arrays; 10,000 wells in each block correspond to the 10^6^ microwells on a single fabricated device. Phase-contrast image and fluorescent image of a block of well array are shown. Scale bars, 1 mm

The principle of our idICA is illustrated in Fig. 1A, B. EVs were captured on cholera toxin B subunit (CTB)-conjugated magnetic beads that specifically recognize GM1 [21] on EVs; EVs were then identified with an antibody against target proteins on the EV surface, such as the EV marker CD9 [22] or Aß, which form a sandwich immunocomplex on those beads. The detection antibody was labeled with the DNA oligonucleotide IL28t or IL28c [13]. The resultant complexes were loaded into our digital device followed by an injection of substrates for the invasive cleavage assay (ICA) and were enclosed with fluorinated oil; fluorescence signals emitted only at microwells with the filled EV–bead–antibody complex were analyzed. As illustrated in Fig. 1C, the ICA utilizes a Flap endonuclease 1 called FEN-1to cleave substrates containing generic unannealed 5′-flap structures in the detection reaction. In the primary reaction, when invasive oligonucleotides and probe oligonucleotides hybridize to target DNA conjugated to detection antibodies in the microwells, 5′-flap structures in the probe oligonucleotides are generated and cleaved by FEN-1. At the optimal temperature (66°C), the invasive oligonucleotides remain annealed while the probe oligonucleotides undergo dissociation and reassociation with the targets, resulting in the accumulation of cleaved 5′-flaps. Afterward, the cleaved 5′-flaps participate in a second reaction as invasive oligonucleotides. They bind to secondary reaction template oligonucleotides, forming other 5′-flap structures. Fluorescence signals are emitted when the 5′-flaps of the fluorescence probes are cleaved by FEN-1 to induce the fluorophores detaching from the quencher molecules. Using cyclo-olefin polymers (COPs), we developed a microwell array device that has 10,000 reactors (Fig. 1D). This fabricated device, which has an extensive array of hydrophilic-in-hydrophobic structures substrate, is assembled with CYTOP-coated glass with an access port for sample injection. An array of CYTOP through-hole structures (diameter, 5 mm; thickness, 3 mm; center-to-center distance, 10 mm) was fabricated on a glass substrate. In this system, an individual bead was filled into a femtomolar droplet array. Each droplet, which contained a bead, was categorized according to a fluorescence-based binary scale, with “1” being signal-emitting droplets and “0” the droplet without a signal. Fluorescent droplets with numbers “1” and “0” were counted, and the ratio of their fluorescence to beads was calculated as the EV concentration.

**Fig. 2:**
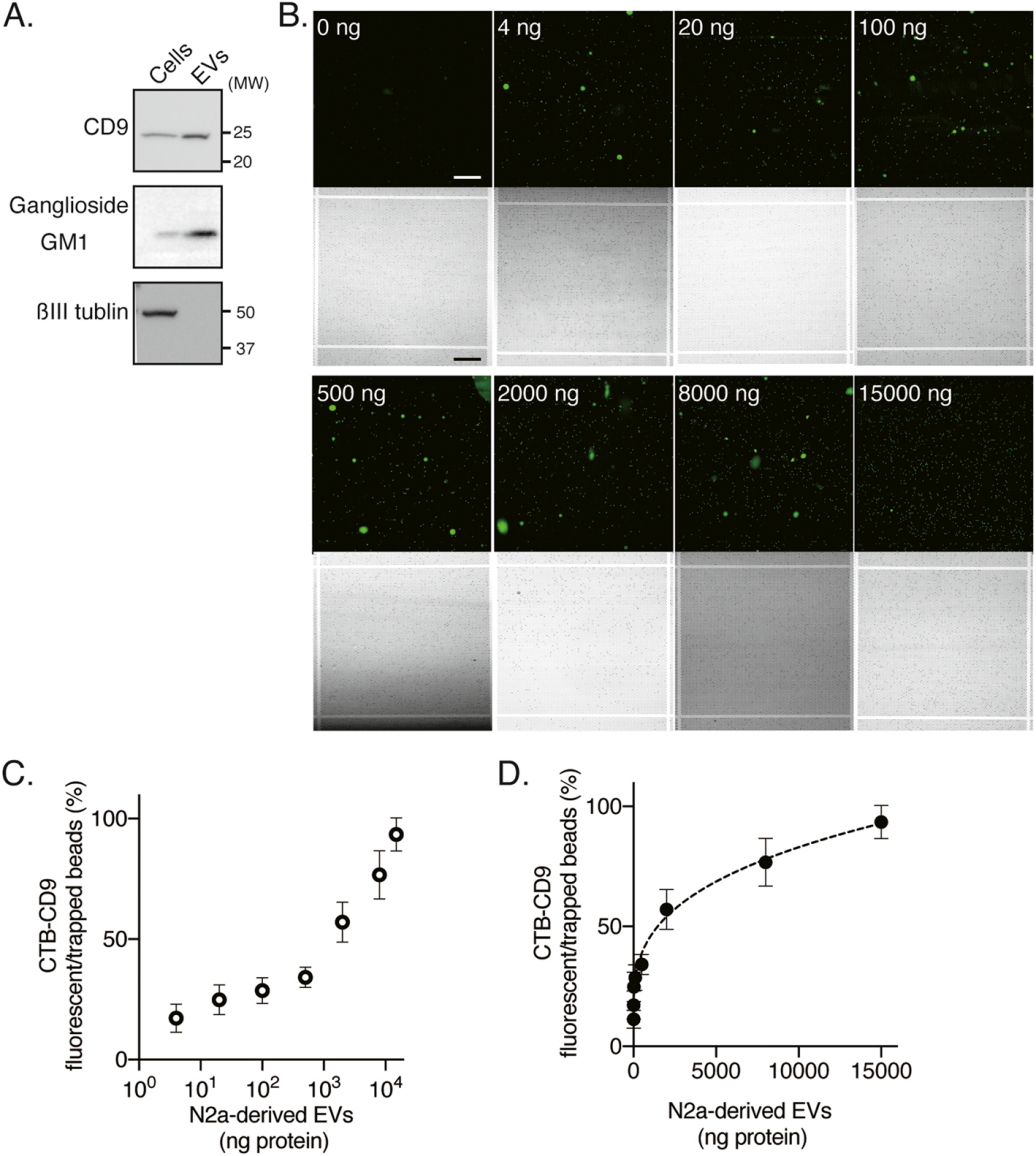
Quantification of GM1-containing EVs Using the idICA. **A** Western blot analysis of CD9, ganglioside GM1, and ßIII tubulin in N2a cell lysates (1 × 10^5^ cells/lane) and EVs (1 × 10^7^ cells/lane). **B** Representative fluorescent images of various concentrations of N2a-derived EVs in the idICA, which is constructed from CTB capture and anti-CD9 detection. Each image shows a block of well array corresponding to 10,000 microwells. Scale bar, 200 μm. **C**, **D** The ratio of fluorescent beads to trapped beads in a block of well array is plotted as the concentration of CD9 captured on CTB-coated beads (CTB-CD9) in N2a-derived EVs. Plots on the semi-logarithmic (**C**) and linear (**D**) scales are shown. Data represent mean ± SD (*n* = 3 each)

We first assessed the idICA system for quantification of GM1-containing EVs. Ultracentrifugation was used to isolate EVs from the culture supernatant of mouse neuroblastoma N2a cells. Electron microscopy confirmed that the purified EVs consisted mainly of small membrane vesicles (Fig. S1A), similar to those previously described [23]; a nanoparticle analyzer verified their sizes, which ranged from 60 to 140 nm (Fig. S1B). Western blotting revealed that ganglioside GM1, not ßIII tubulin expression, was similar to the EV marker CD9; GM1 and CD9 were substantially more abundant in the EV fractions than in the cell lysates (Fig. 2A). We, therefore, used CTB to capture GM1 on EVs and anti-CD9 antibody to detect GM1-containing EVs. Fluorescence images were used to determine whether the beads had GM1-containing EVs. Representative fluorescence images of an array block corresponding to 10,000 microwells at different N2a-derived EV-concentrations are shown in Fig. 2B. The ratios of fluorescent beads to trapped beads are plotted on semi-logarithmic (Fig. 2C) and linear (Fig. 2D) scales as the concentration of GM1-containing EVs in N2a-derived EVs; these data indicated a proportional increase in a dynamic range from 0 to 15,000 ng protein.

**Fig. 3:**
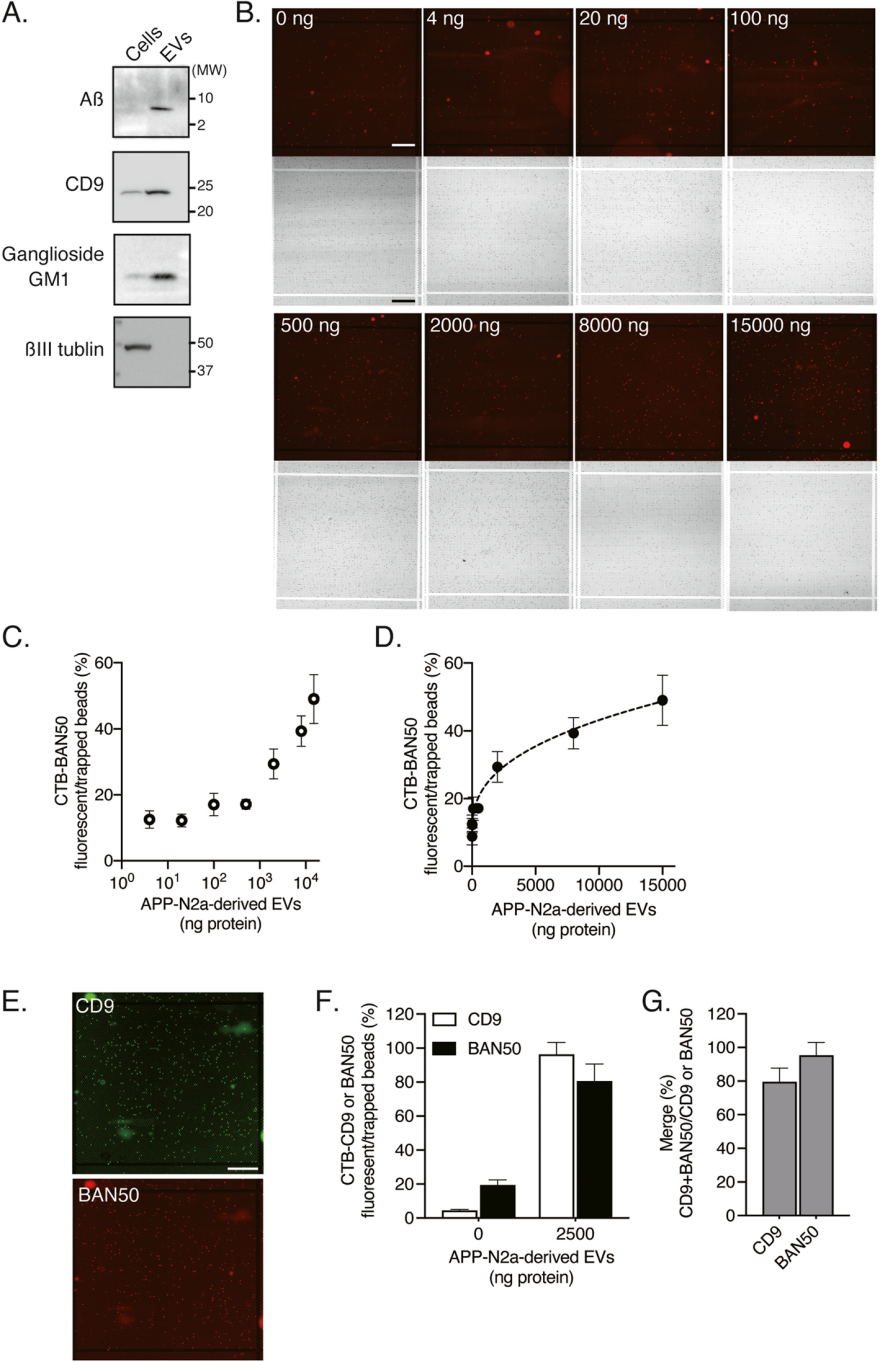
Quantification of Aß-bound and GM1-containing EVs using the idICA. **A** Western blot analysis of Aß, ganglioside GM1, and ßIII tubulin in APP-N2a cell lysates (1 × 10^5^ cells/lane) and EVs (1 × 10^7^ cells/lane). **B** Representative fluorescent images of various concentrations of APP-N2a-derived EVs in the idICA, which is constructed from CTB capture and anti-Aß detection. Each image displays a block of well array corresponding to 10,000 microwells. Scale bar, 200 μm. **C**, **D** The ratio of fluorescent beads to trapped beads in a block of well array is plotted as the concentration of Aß captured on CTB-coated beads (CTB-BAN50) in APP-N2a-derived EVs. Plots on the semi-logarithmic (**C**) and linear (**D**) scales are shown. Data represent mean ± SD (*n* = 3 each). **E** Representative images of APP-N2a-derived EVs (2500 ng protein) in the double color idICA using anti-CD9 antibody and BAN50. Each image displays a block of well array corresponding to 10,000 microwells. Scale bar, 200 μm. **F** The ratio of BAN50 or CD9 fluorescent beads to trapped beads. **G** The overlap rate between BAN50 and CD9 fluorescent beads. Data represent mean ± SD (*n* = 5 each)

Next, we validated the idICA system for quantifying Aß-bound and GM1-containing EVs. Western blot analysis showed that Aß and ganglioside GM1, but not ßIII tubulin, were highly expressed in EVs isolated from the culture supernatant of APP-N2a cells by ultracentrifugation (Fig. 3A). Representative fluorescence images of an array block with various concentrations of APP-N2a-derived EVs are shown in Fig. 3B; CTB and an anti-Aß antibody were used to capture GM1-containing and Aß-bound EVs. Semi-logarithmic (Fig. 3C) and linear (Fig. 3D) graphs show the ratio of fluorescent beads to trapped beads, plotted as the concentration of Aß-bound and GM1-containing EVs. The dynamic range for the digital detection of Aß on GM1-containing EVs increased from 0 to 15,000 ng protein. To verify whether the fluorescence signals are specific for Aß in the EVs, we compared the signals of Aß between Aß-bound EVs that were derived from APP-N2a cells and non-Aß-bound EVs that were derived from HEK293 cells. The dynamic range for the digital detection of Aß on GM1-containing EVs increased from 0 to 2500 ng protein in the EVs derived from APP-N2a cells, but not those derived from HEK293 cells (Fig. S3). We conducted a multi-color idICA with anti-CD9 antibody and an anti-Aß antibody (BAN50) to capture GM1-containing and Aß-bound EVs in the APP-N2a EVs. Representative fluorescence images of CD9 and Aß in an array block are shown in Fig. 3E. The ratios of CD9 or BAN50 fluorescent beads to trapped beads, and the merged BAN50 and CD9 fluorescent beads to individual fluorescent beads are plotted (Fig. 3F, G). These data indicated that the multi-color idICA successfully assessed GM1-containing and Aß-bound EVs in APP-N2a-derived EVs. The fluorescent beads of BAN50 were almost completely merged with CD9, demonstrating Aß-bound and GM1-containing fluorescence were originated from the EVs.

**Fig. 4:**
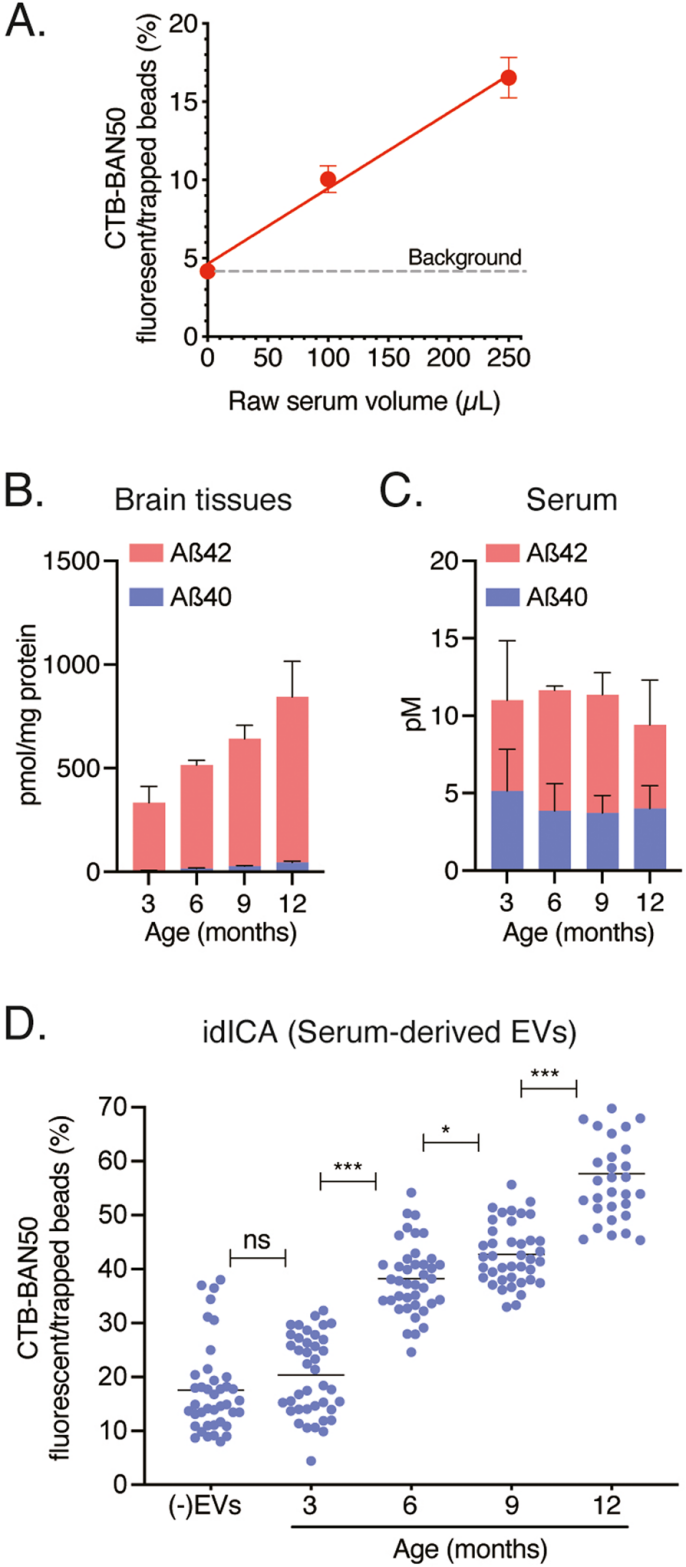
Quantification of Aß-bound and GM1-containing EVs in sera of APP Tg mice using the idICA. **A** The ratio of fluorescent beads to trapped beads in a block of well array is plotted as the concentration of Aß captured on CTB-coated beads (CTB-BAN50) in the indicated volume of APP Tg mouse serum-derived EVs. Plots on a linear scale are shown. Data represent mean ± SD (*n* = 3 each). **B**, **C** The levels of Aβ_1-40_ and Aβ_1-42_ in brain tissues (**B**) and sera (**C**) of APP Tg mice were measured by ELISA (*n* = 4 animals per age group). **D** The ratio of fluorescent beads to trapped beads in a block of well array is plotted as the concentration of Aß captured on CTB-coated beads (CTB-BAN50) in 100 μL of 3-, 6-, 9-, and 12-month-old APP Tg mouse serum-derived EVs. Data represent mean ± SD. (-)EVs represents PBS only; serum-derived EVs from 3-, 6-, 9-, and 12-month-old mice, *n* = 4-5 each; each dot represents the ratio of fluorescent beads to trapped beads in a block of well array and each EV is determined in eight blocks of well array (*P < 0.05; ***P < 0.001 by one-way ANOVA)

Human serum, which is enriched with EVs secreted by cells from various tissues and organs, is a suitable source for liquid biopsy [23]. Brain-derived EVs are present in the peripheral blood circulation and are potential biomarkers for monitoring the pathophysiology of central nervous system diseases [24, 25]. Using our idICA, we quantified GM1-containing and Aß-bound EVs in purified EVs from the sera of APP Tg mice. The EVs derived from APP Tg mouse serum were confirmed by electron microscopy, nanoparticle analyzer, and Western blotting, which demonstrated that the EVs were small membrane vesicles 50–110 nm in diameter (Fig. S4A, B) with ganglioside GM1 and the EV marker CD9 as well as Aß (Fig. S4C). GM1-containing and Aß-bound EVs in APP Tg mouse serum up to 250 μL were quantitatively detected (Fig. 4A). Further studies are required to determine whether GM1-containing and Aß-bound EVs are associated with age-dependent amyloidopathy in AD model mice. Aß_1-40_ and Aß_1-42_ levels were measured in the brain tissues and sera of APP Tg mice with traditional ELISA. Aß_1-40_ and Aß1_-42_ levels were measured in the brain tissues and sera of 3-, 6-, 9-, and 12-month-old APP Tg mice with traditional ELISA and immunohistochemistry. Age-dependent increase in Aß levels, corresponding to increased Aß deposition, were found in the brain tissues of APP Tg mice (Fig. 4B); conventional ELISA, however, did not detect any changes in Aß_1-40_ and Aß_1-42_ levels in whole sera from these mice (Fig. 4C). Aß levels in the serum-derived EVs were below the detection limit of conventional ELISA (Fig. S4E). Using the idICA, we successfully evaluated GM1-containing and Aß-bound EVs in serum-derived EVs from of 3-, 6-, 9-, and 12-month-old APP Tg mice; the concentration of Aß captured on CTB-coated beads was increased in an age-dependent manner (Fig. 4D, Table S1). Our idICA quantitatively detected the levels of Aß-bound and GM1-containing EVs in APP Tg mouse sera, demonstrating the age-dependent increase of GM1-containing and Aß-bound EVs in mouse sera was in accordance with Aß levels in mouse brains.

## Discussion

By 2050, the number of people with dementia is estimated to be over 131 million worldwide, of which AD may account for 50–80% of all cases of dementia [26, 27]. The discovery of non-invasive and easy-to-use biomarkers to monitor brain amyloid pathogenesis is an urgent challenge for the early diagnosis and prediction of AD. In this study, we developed a droplet-based digital assay system to measure Aß-bound and GM1-containing EVs (idICA); we found a positive correlation between Aß-bound EVs in mice sera and Aß burden in the aged APP Tg mouse brain, indicating that it may be a valuable biomarker candidate for brain amyloid pathology.

Our idICA used CTB-labeled paramagnetic beads to selectively capture ganglioside GM1-containing EVs. Gangliosides are glycosphingolipids composed of a ceramide attached to a sugar chain with one or more sialic acid residues. Simple gangliosides, such as GM3, predominate in most peripheral tissues; however, over 90% of brain gangliosides are composed of four complex gangliosides, GM1, GD1a, GD1b, and GT1b [28, 29]. EVs, particularly exosomes, contain enriched lipid raft-resident molecules, including gangliosides [11, 30]. The origin of EVs has not yet been identified; however, most EVs detected in blood by our idICA are presumed to be GM1-containing brain-derived EVs. It is also important to note that PS-deficient EVs, which have been reported as blood EV subpopulations [31], were excluded in this idICA study since only PS-exposing EVs were purified by MagCapture isolation system.

Our idICA quantitatively detected the levels of Aß-bound and GM1-containing EVs in APP Tg mouse sera, demonstrating the age-dependent increase of GM1-containing and Aß-bound EVs in mouse sera was in accordance with Aß levels in mouse brains. GM1 and the exosome marker protein Alix are abundant in amyloid plaques in human brain [7, 32]. Aß recognizes GM1 clusters in cholesterol-enriched microdomains (lipid rafts); the resulting ganglioside–Aß complexes act as a seed to accelerate amyloid fibril or oligomer formation [4, 32, 33]. We previously showed that ganglioside-enriched EVs are involved in Aß clearance by transporting Aß into microglia in vitro and in vivo [9, 10]. Sphingomyelinase inhibition reduces EV release and amyloid plaque load in AD model mice [12, 34]. Aß-associated EVs may aid Aß accumulation and amyloid plaque formation caused by an excess of EVs or the absence of glial phagocytic activities, whereas free Aß-bound EVs leak into the peripheral circulation. Western blotting revealed that only Aß was detected by BAN50 in the APP-N2a-derived EVs and the serum-derived EVs (Figs. S2B and S5), demonstrating that the target of BAN50 in the EVs was Aß species, but not full-length APP and APP fragments such as APP C-terminal fragments. The form of the Aß associated with EVs has not been identified; however, they may include monomeric, oligomeric, and fibrillar conformations of Aß [9, 35], all of which are recognized by the detection antibody BAN50 in the idICA system.

Several complicated methods are used to measure Aß in blood; to predict amyloid plaque formation in the brain, mass spectrometry is used to identify Aß isoforms such as APP669-711 and Aß_1-40_ [36–38]. A further approach for the quantitative analysis and molecular profiling of exosomes in biofluid also involves an EV immunoassay method with specialized setups of surface plasmon resonance-based nPLEX and iMEX platforms [39, 40]. The idICA that enables accurate (femtogram) quantification of nucleic acids and proteins was recently developed [41]. This system uses a high precision-manufactured microwell device to provide up to 1000 times greater sensitivity than a traditional idICA [13]. Our idICA was developed to rapidly and easily detect nanogram concentrations of single Aß-bound and GM1-containing EVs; it can be used, in advance, to monitor Aß load in the brain for the assessment of blood-based pathologic and clinical diagnosis of AD.

As shown in Fig. 3, the idICA system can use a multi-color detection approach to simultaneously measure multiple proteins. A recent proteomic analysis shows that specific EV proteins are altered during AD progression [42, 43]. Aß, combined with other AD biomarkers, such as tauopathy, inflammation, or neuronal degeneration, may be used to further classify the pathological stages in AD. This system will pave the way for providing a minimally invasive approach for identifying risk factors for AD onset and effective disease-modifying therapies during the preclinical stages of the disease.

## Limitations

The major limitation is that our idICA is needed to analyze human clinical samples further to demonstrate the relationship between peripheral Aß-associated EVs and brain Aß burdens in the Alzheimer’s continuum. Glycolipid GM1 are identical among species, making the idICA possible to detect EVs in human subjects. In addition, as the AD model mice used in this study express a familial mutant form of APP and show progressive amyloid depositions during the short term, we need to verify the availabilities of the EVs as a biomarker in sporadic AD.

## Conclusion

In the present study, we developed a digital detection system idICA to analyze Aß-associated and ganglioside GM1-contained EVs in AD model mouse sera and found that peripheral EVs harboring Aß and GM1 in blood reflect Aß burdens in mouse brains. The idICA is an easy and valuable tool for quantitative detection of EVs as a blood biomarker for preclinical AD diagnosis.

## Supplementary Information

**Additional file 1: Supplemental Fig. 1.** (A) Representative image of N2a-derived EVs using electron microscopy. (B) Size distribution of N2a-derived EVs analyzed by a nanoparticle analyzer, qNano.

**Additional file 2: Supplemental Fig. 2.** Uncropped membrane images of western blot analysis. (A) N2a cells and N2a-derived EVs. (B) APP-N2a cells and APP-N2a cells-derived EVs.

**Additional file 3: Supplemental Fig. 3.** The ratio of fluorescent beads to trapped beads as the concentration of Aß captured on CTB-coated beads (CTB-BAN50) in APP-N2a cells- and HEK293 cells-derived EVs.

**Additional file 4: Supplemental Fig. 4.** (A) Size distribution of APP Tg serum-derived EVs analyzed by a nanoparticle analyzer, qNano. (B) Representative image of APP Tg serum-derived EVs by electron microscopy. (C) Western blot analysis of Aß, ganglioside GM1, CD9, and ßIII tubulin in APP Tg serum-derived EVs. (D) Representative images of hippocampal sections immunostained with Aβ. Scale bars, 200 μm. (E) The levels of Aβ_1-40_ and Aβ_1-42_ in whole sera and serum-derived EVs of 12-month-old APP Tg mice were measured by conventional ELISA (*n* = 4 each). n.d., not detected.

**Additional file 5: Supplemental Fig. 5.** Uncropped membrane images of western blot analysis of the APP Tg serum-derived EVs.

**Additional file 6: Supplementary Table 1.** Raw data of Fig. 4d.

## Data Availability

Not applicable.
