# Supplementary figures and images for: Immuno-digital invasive cleavage assay for analyzing Alzheimer’s amyloid ß-bound extracellular vesicles
Source: Alzheimers Res Ther. 2022 Oct 3;14:140. doi: 10.1186/s13195-022-01073-w (PMC9528138; doi:10.1186/s13195-022-01073-w)

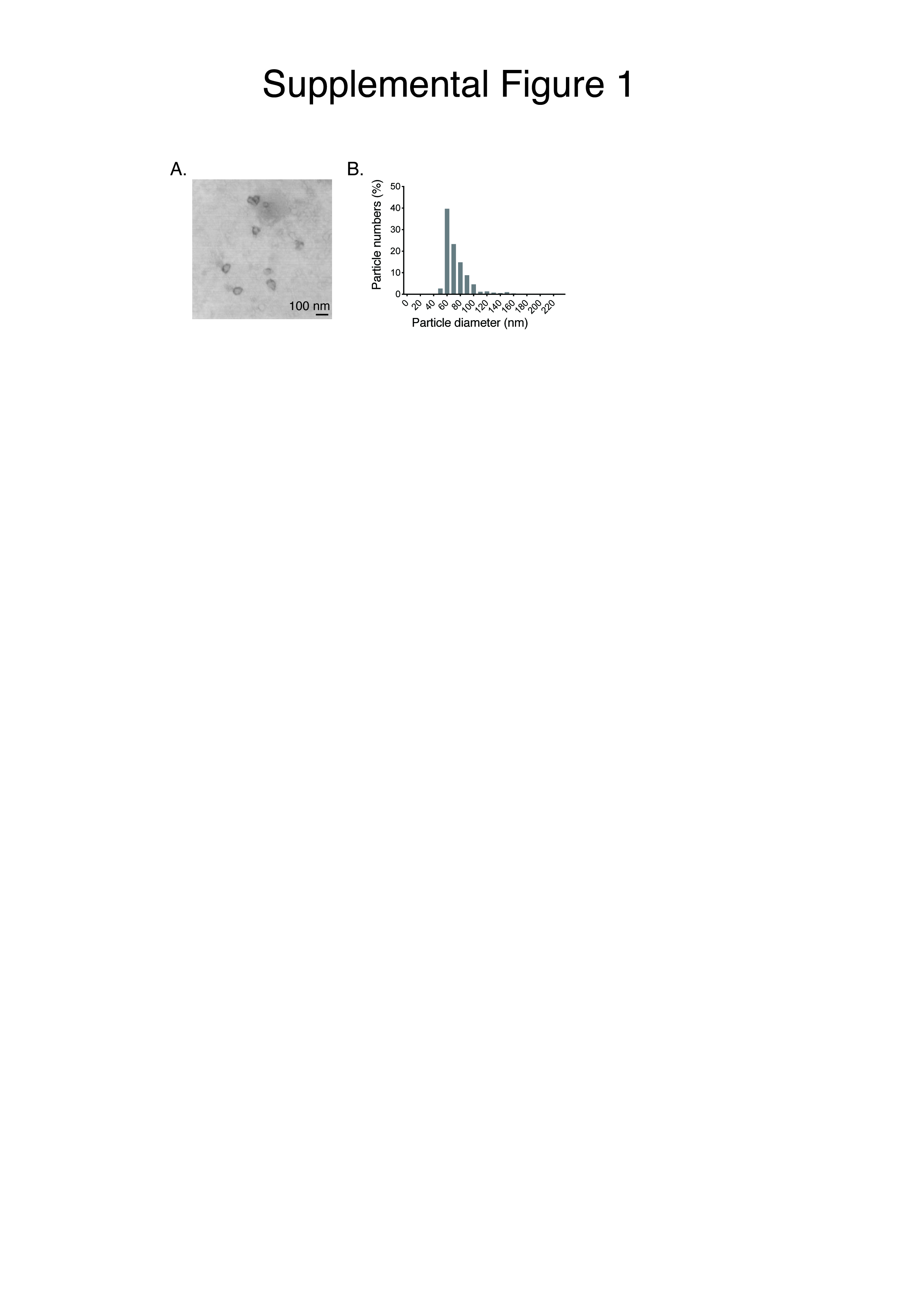

Supplement: Supplementary file 1 — Additional file 1: Supplemental Fig. 1. (A) Representative image of N2a-derived EVs using electron microscopy. (B) Size distribution of N2a-derived EVs analyzed by a nanoparticle analyzer, qNano. [file 13195_2022_1073_MOESM1_ESM.jpg]

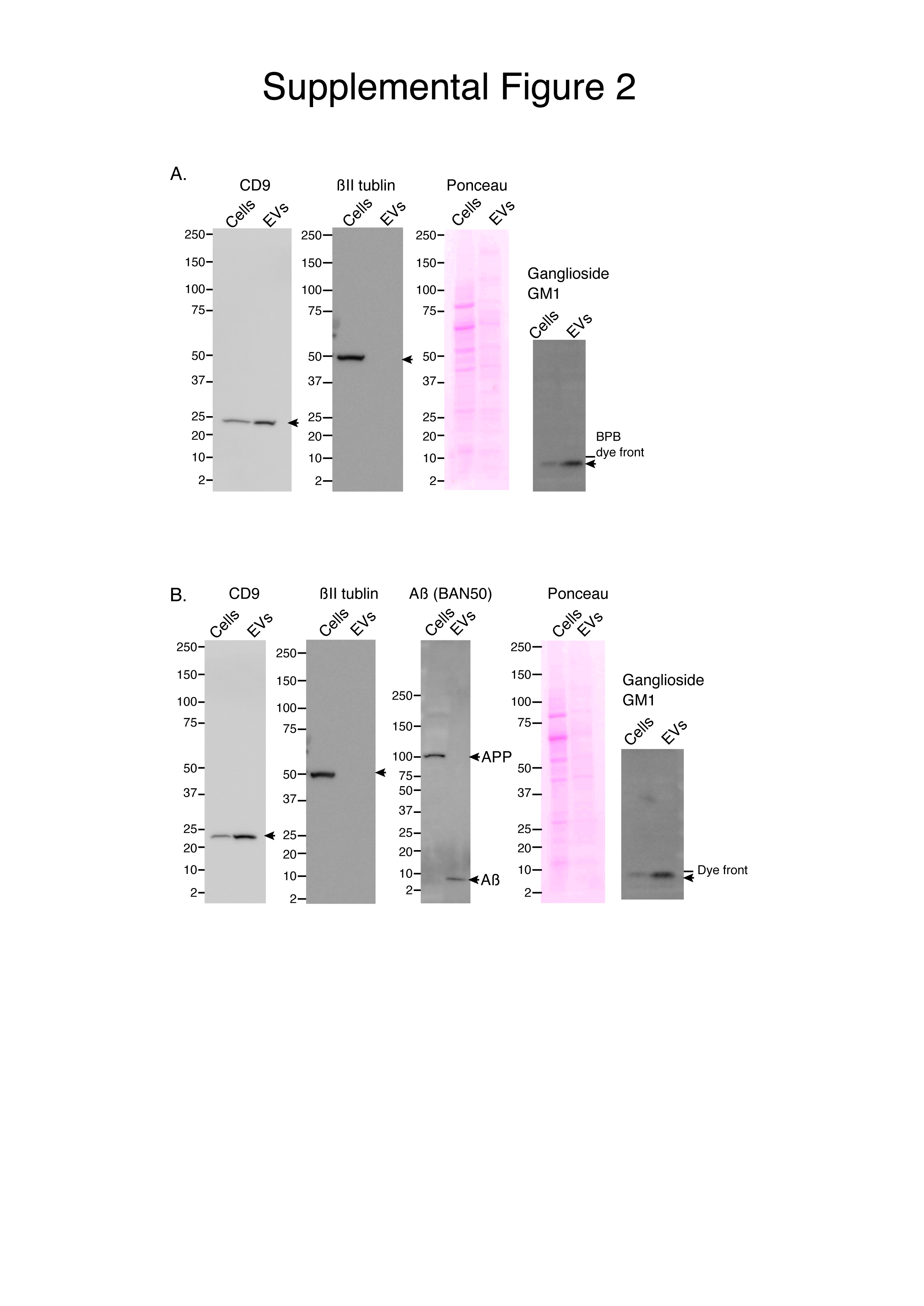

Supplement: Supplementary file 2 — Additional file 2: Supplemental Fig. 2. Uncropped membrane images of western blot analysis. (A) N2a cells and N2a-derived EVs. (B) APP-N2a cells and APP-N2a cells-derived EVs. [file 13195_2022_1073_MOESM2_ESM.jpg]

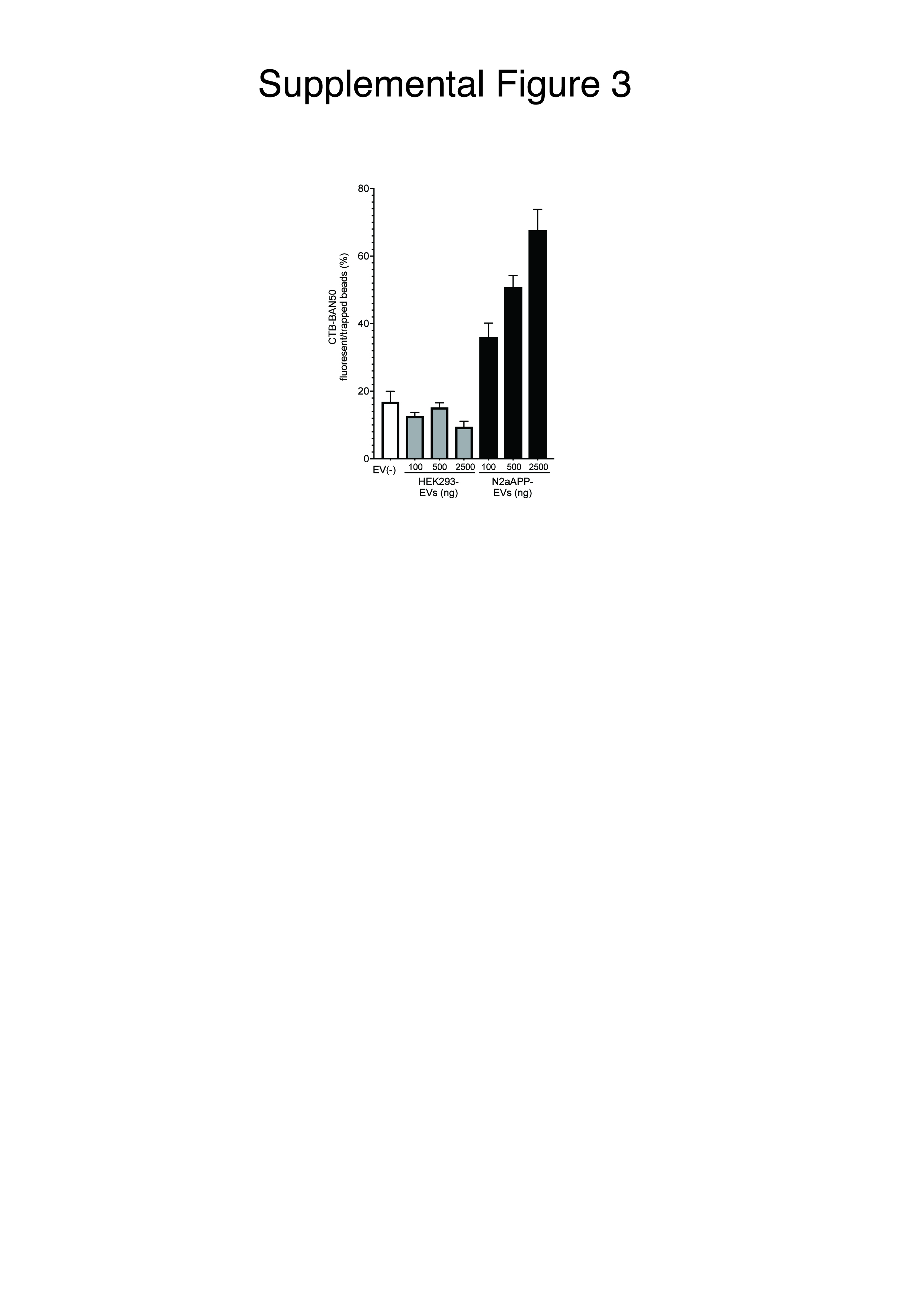

Supplement: Supplementary file 3 — Additional file 3: Supplemental Fig. 3. The ratio of fluorescent beads to trapped beads as the concentration of Aß captured on CTB-coated beads (CTB-BAN50) in APP-N2a cells- and HEK293 cells-derived EVs. [file 13195_2022_1073_MOESM3_ESM.jpg]

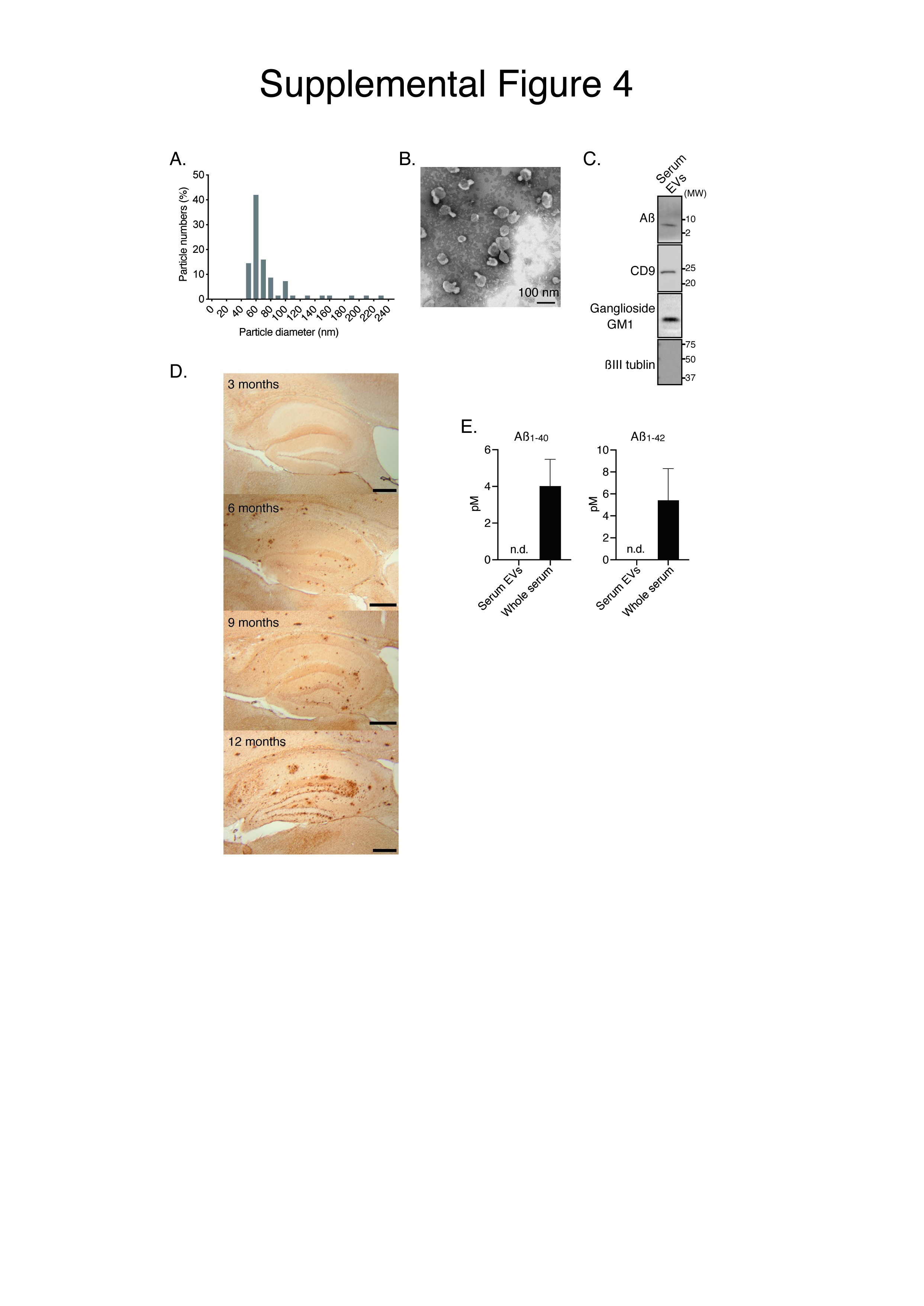

Supplement: Supplementary file 4 — Additional file 4: Supplemental Fig. 4. (A) Size distribution of APP Tg serum-derived EVs analyzed by a nanoparticle analyzer, qNano. (B) Representative image of APP Tg serum-derived EVs by electron microscopy. (C) Western blot analysis of Aß, ganglioside GM1, CD9, and ßIII tubulin in APP Tg serum-derived EVs. (D) Representative images of hippocampal sections immunostained with Aβ. Scale bars, 200 μm. (E) The levels of Aβ1-40 and Aβ1-42 in whole sera and serum-derived EVs of 12-month-old APP Tg mice were measured by conventional ELISA (n = 4 each). n.d., not detected. [file 13195_2022_1073_MOESM4_ESM.jpg]

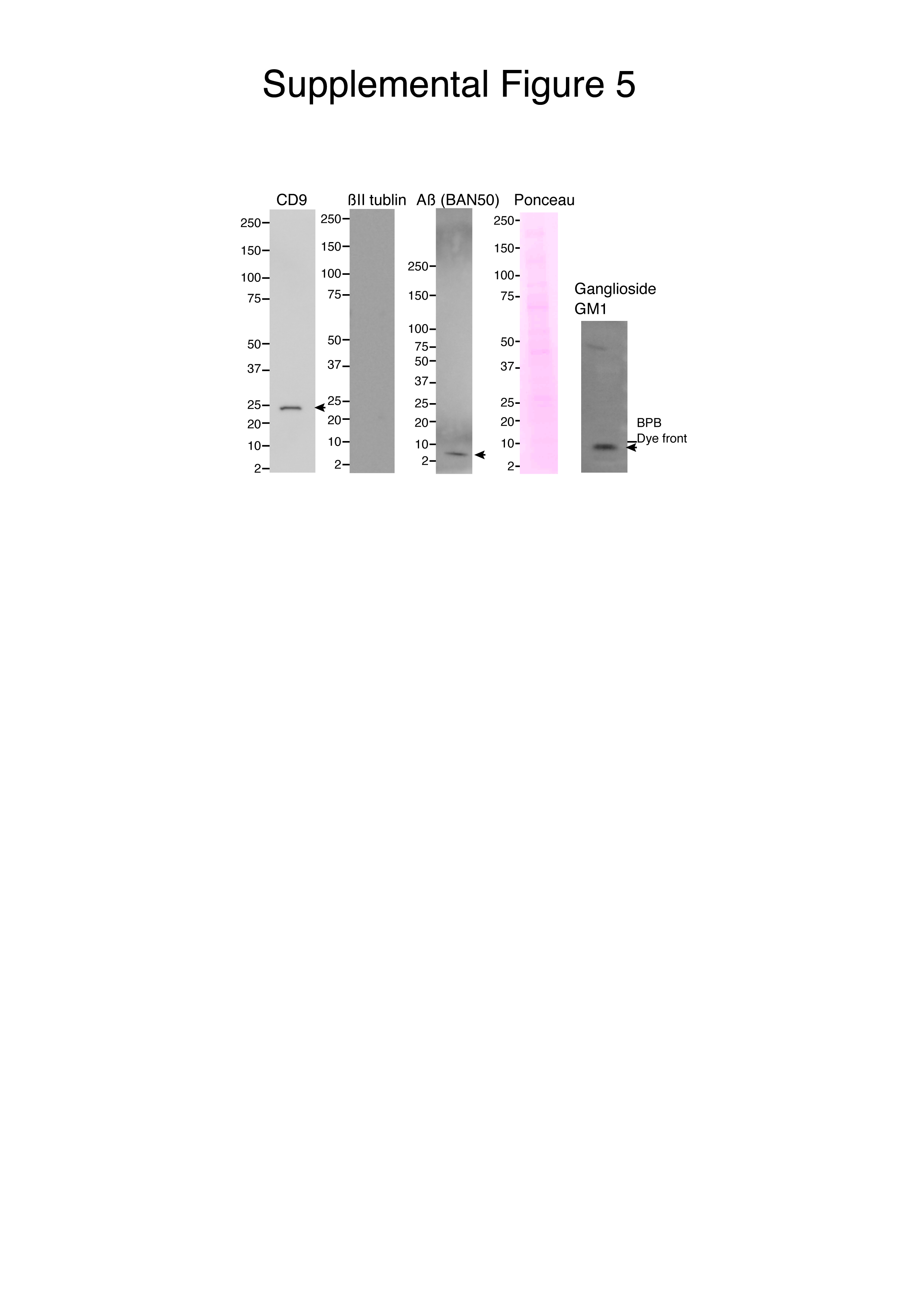

Supplement: Supplementary file 5 — Additional file 5: Supplemental Fig. 5. Uncropped membrane images of western blot analysis of the APP Tg serum-derived EVs. [file 13195_2022_1073_MOESM5_ESM.jpg]
